# Editorial: Advances in translational and applied stem cell biology

**DOI:** 10.3389/fcell.2024.1489085

**Published:** 2024-09-16

**Authors:** Fangfang Zhu, Yan-Ru Lou, Cheguo Cai, Jianjun Zhou

**Affiliations:** ^1^ School of Biomedical Engineering, Shanghai Jiao Tong University, Shanghai, China; ^2^ HemaCell Biotechnology Inc., Suzhou, China; ^3^ Faculty of Pharmacy, University of Helsinki, Helsinki, Finland; ^4^ Hangzhou Institute for Advanced Study, University of Chinese Academy of Sciences, Hangzhou, China; ^5^ Research Center for Translational Medicine, Cancer Stem Cell Institute, Shanghai East Hospital, School of Medicine, Tongji University, Shanghai, China

**Keywords:** stem cell, cancer stem cell, stem cell-based therapy, repeat-dose toxicity, translational stem cell biology, applied stem cell biology

Stem cells play a vital role in embryo-fetal development and the maintenance of adult tissue homeostasis, possessing the ability to replace damaged cells and modulate immune responses. Over recent decades, substantial progress in stem cell research has demonstrated their potential to restore organ function, promote tissue regeneration, and develop innovative therapeutic strategies for a wide range of diseases. The Research Topic “*Advances in Translational and Applied Stem Cell Biology*” gathers the latest developments in this field, with a focus on bridging the gap between basic research and clinical applications.

Stem cells are characterized by their capacities for self-renewal, multi-directional differentiation, rapid proliferation, and relatively low immunogenicity. These properties are key to tissue repair and regeneration, positioning stem cells as a promising therapeutic approach. A long-standing area of focus has been the repair of neurological damage, particularly in the context of stroke treatment. The high disability and mortality rates associated with ischemic stroke make it a serious threat to human health, often leading to significant neurological deficits. Cha et al provide a comprehensive overview of the role of stem cells in ischemic stroke, highlighting their potential as therapeutic interventions. The review summarizes various stem cell therapies that enhance recovery through mechanisms such as regulating autophagy, promoting neuroprotection and regeneration, reestablishing blood supply, repairing the blood-brain barrier, and exerting anti-inflammatory, immunomodulatory, and paracrine effects.

While stem cells are central in regenerative medicine, their application extends beyond this scope. In cancer treatment, the focus shifts towards targeting cancer stem cells (CSCs) to inhibit tumor growth and metastasis. Su et al review the latest advances in the identification and characterization of prostate cancer stem cells (PCSCs) and their role in tumor relapse and disease progression. The review also outlines therapeutic strategies targeting PCSCs, including PCSC-related pathway therapy, miRNA therapies, and immunotherapies. These insights into PCSC biology and targeted treatments hold promise for optimizing therapies for prostate cancer, underscoring the importance of CSCs in oncology.

Despite progress in stem cell research, current laboratory-scale protocols for cultivating stem cells are insufficient to meet the demands of clinical applications. Significant challenges remain in scaling up stem cell production processes, but three-dimensional (3D) culture systems offer a promising solution for large-scale production, addressing the substantial quantity requirements. Yuan et al develop a scalable expansion method for human pluripotent stem cells (hPSCs) using a 3D culture system supplemented with human platelet lysate (hPL). As a xeno-free and serum-free supplement, hPL shows potential for broad application across culture systems. With hPL supplementation, hPSCs demonstrated enhanced proliferation, higher cell viability, and reduced intercellular variability. Importantly, after prolonged culture in the hPL-supplemented 3D culture system, hPSCs maintained pluripotency marker expression, the capacity to differentiate into all three germ layers, and a normal karyotype, confirming the practicability and safety of hPL supplementation.

While a robust *in vitro* culture system ensures a steady source of stem cells, certain therapeutic applications require fully mature, functional, and safe cell types for replacement therapy. This underscores the critical importance of scalable differentiation and stringent quality control technologies. For instance, liver transplantation remains the main reliable treatment for patients with end-stage liver disease, yet access to a sufficient number of hepatocytes is limited by scarcity of human liver tissue. Thus, harnessing the therapeutic potential of stem cells as an alternative source of mature hepatocytes is crucial for overcoming these limitations. Tapparo et al develop a 3D rotary *in vitro* culture system to generate functional hepatocyte-like cells from Human Liver Stem Cells (HLSCs) and employed a matrix multi-assay approach to comprehensively characterize HLSC differentiation and ensure cell quality. Hepatocytes differentiated in this system exhibited upregulated expression of hepatic markers, increased urea and FVIII secretion, and improved functionality, as evidenced by an optimized indocyanine green *in vitro* assay. These advancements in 3D culture systems and comprehensive quality control technologies promote the standardization of hepatocyte production, facilitating their clinical application.

The development of stem cell expansion and differentiation systems has made stem cell therapy feasible, while comprehensive identification assays ensure the quality of stem cell products. However, rigorous safety assessments remain essential for analyzing adverse drug reactions and supporting the clinical translation of stem cell-based therapies. Pan et al explore the general toxicity, immune perturbation, and toxicokinetics of human umbilical cord mesenchymal stem cell (hUC-MSC) injection in cynomolgus monkeys. Their findings demonstrated the safety of repeated hUC-MSC doses administered via intravenous and subcutaneous routes. No systemic toxicity was observed in key parameters like weight, temperature, hematology, clinical chemistry, ECG, and pathology. However, infusion-related reactions such as coma and decreased respiratory rate were observed at high doses during intravenous administration. These findings underscore the importance of safety validation and highlight the critical role of non-clinical safety evaluations in guiding clinical applications.

In summary, significant strides have been made in stem cell research, particularly in neurological repair and prostate cancer treatment. As we move closer to clinical applications, the focus must remain on the large-scale *in vitro* culture, expansion, and differentiation of stem cells while ensuring rigorous quality control throughout the process. Additionally, the safety of stem cell therapies, particularly the potential toxicity of adoptive cell therapies, requires thorough evaluation. Advances in these areas will be crucial for fully realizing the therapeutic potential of stem cells, paving the way for safer and more effective treatments for various diseases. The Research Topic of studies in this Research Topic will certainly facilitate the translation of basic stem cell research and technology into clinical applications.

